# CAT: a conditional association test for microbiome data using a permutation approach

**DOI:** 10.1093/bib/bbaf326

**Published:** 2025-07-11

**Authors:** Yushu Shi, Liangliang Zhang, Kim-Anh Do, Robert R Jenq, Christine B Peterson

**Affiliations:** Department of Population Health Sciences, Weill Cornell Medicine, 575 Lexington Avenue, New York, NY 10065, United States; Department of Population and Quantitative Health Sciences, Case Western Reserve University, 2103 Cornell Road, Cleveland, OH 44106, United States; Department of Biostatistics, University of Texas MD Anderson Cancer Center, 7007 Bertner Avenue, Houston, TX 77030, United States; Department of Hematology & Hematopoietic Cell Transplantation, City of Hope, 1500 East Duarte Road, Duarte, CA 91010, United States; Department of Biostatistics, University of Texas MD Anderson Cancer Center, 7007 Bertner Avenue, Houston, TX 77030, United States

**Keywords:** beta diversity metrics, permutation, conditional association test, coefficient of determination, microbiome data

## Abstract

In microbiome analysis, researchers often seek to identify taxonomic features associated with an outcome of interest. However, microbiome features are intercorrelated and linked by phylogenetic relationships, making it challenging to assess the association between an individual feature and an outcome. This paper proposes a novel conditional association test, **CAT**, that can account for other features and phylogenetic relatedness when testing the association between a feature and an outcome. **CAT** adopts a permutation approach, measuring the importance of a feature in predicting the outcome by permuting operational taxonomic unit/amplicon sequence variant counts belonging to that feature from the data and quantifying how much the association with the outcome is weakened through the change in the coefficient of determination $R^{2}$. Compared with marginal association tests, it focuses on the added value of a feature in explaining outcome variation that is not captured by other features. By leveraging global tests including PERMANOVA and MiRKAT-based methods, **CAT** allows association testing for continuous, binary, categorical, count, survival, and correlated outcomes. We demonstrate through simulation studies that **CAT** can provide a direct quantification of feature importance that is distinct from that of marginal association tests, and illustrate **CAT** with applications to two real-world studies on the microbiome in melanoma patients: one examining the role of the microbiome in shaping immunotherapy response, and one investigating the association between the microbiome and survival outcomes. Our results illustrate the potential of **CAT** to inform the design of microbiome interventions aimed at improving clinical outcomes.

## Introduction

In recent years, the advent of next-generation sequencing technologies has revolutionized microbiome research, allowing for unprecedented detail in the characterization of microbial communities and paving the way for advanced analytic approaches. By providing high-resolution profiles of the human microbiome, these techniques have prompted the development of numerous computational methods designed to leverage microbial composition data for more accurate disease outcome predictions [1–3]. Despite these breakthroughs, analyzing microbiome data remains challenging due to its high dimensionality and the structural relatedness of the observed features. As a starting point in assessing the link between the microbiome and an outcome, researchers often test the global association of a phenotype with the microbiome as a whole. Global association tests addressing this question typically employ microbiome-specific metrics (also called beta diversity measures) that are more robust to extreme values than classical Euclidean distances. Popular choices include Bray–Curtis dissimilarity [4], which is designed for count data, and weighted and unweighted UniFrac [5, 6], which incorporate information on the location of features in a phylogenetic tree.

For microbiome datasets with clearly identified sample groups, a popular global association test is PERMANOVA, which utilizes permutation testing to obtain a $P$-value for the null hypothesis that there is no difference in the location of the centroids across groups [7]. PERMANOVA has been widely used in microbial analysis, as it is simple to apply and only requires observations of the outcome variable and the pairwise distances or dissimilarities between samples. Moreover, recent adaptations of PERMANOVA can handle nested designs and correlated outcomes [8].

Another popular global association test for microbiome data is MiRKAT, which is based on kernel machine regression [9]. One advantage of this method is that it can incorporate multiple candidate distance metrics to maximize power for a particular dataset. In addition to continuous and binary response variables, MiRKAT has been extended to handle survival outcomes [10] and correlated or dependent samples [11, 12]. These global association methods have been widely applied in microbiome studies. However, they cannot provide inference on specific taxa. Multiple linear regression, which is commonly used, also cannot be applied here because the sample size is often smaller than the number of features. Existing methods for testing the effect of individual taxa often ignore the presence of related features and focus only on the marginal effect of the individual feature. Simple correlation measures such as Pearson and Spearman capture only the marginal association between a single feature and the outcome. In particular, popular differential abundance methods such as ALDEx2 [13], DESeq2 [14], and ANCOM-BC [15] all adopt a marginal testing framework.

An inherent problem in marginal testing of microbiome data is nested discoveries, where hits are linked within a taxonomic or phylogenetic tree. Taxonomic trees reflect the traditional labeling and organization of microorganisms into groupings such as family or genus, while phylogenetic trees reflect evolutionary history, with branch points corresponding to events that gave rise to differences in the genomic sequences. Both types of trees play a key role in understanding microbiome data. Taxonomic labels serve as a basis for interpretation since they are standardized across studies. Phylogenetic trees are useful in analysis, as they encode rich information on sequence similarity, which drives phenotypic and functional similarity. In Supplemental Figure S6, we present the phylogenetic and taxonomic trees derived from the Gopalakrishnan *et al*. [16] dataset, providing a visual comparison between these two types of trees.

The relatedness among features can make it challenging to pinpoint which taxa play a critical role in influencing outcomes. For example, when a genus is found to be significant, the corresponding higher taxonomic units to which it belongs, such as family and order, also tend to be significant. However, the precise taxonomic level most relevant to the outcome is difficult to establish. A conditional test can provide direct quantification of the importance of a specific feature in contributing information not captured by other features in the data set, addressing a question with potentially greater biological importance than a marginal test.

Notably, the challenge of correlated predictors exists in many high-dimensional datasets, yet is particularly prominent in microbiome data. In other settings, researchers have proposed rigorous definitions of feature-outcome independence. Here, we adopt Candès *et al*.’s [17] definition, where a feature is said to be “null” if and only if the outcome is independent of it conditionally on all other variables.

In this paper, we present a novel conditional test, **CAT**, that provides a natural next step to follow up on a significant global association test result. **CAT** achieves the goal of assessing the importance of individual taxa while accounting for phylogenetic structure and other features in the data set. We refer to this approach as *conditional* to emphasize that the association of each feature is being tested while conditioning on all other features, in contrast to a marginal testing approach, which ignores the presence of related features. More specifically, when examining a selected taxon, the **CAT** test is conditional on the counts of taxa that are not its descendants. This allows **CAT** to focus on the added value of a feature in explaining variation in the outcome not captured by other microbiome features. The remainder of the paper is organized as follows: Section “Approach” describes the proposed **CAT** method in detail. Section “Simulation study” demonstrates its performance using simulated data, and Section “Application to real data” illustrates the method through applications to real datasets with binary and survival outcomes. Section “Concluding remarks” concludes the paper with a discussion.

## Approach

**Figure 1:**
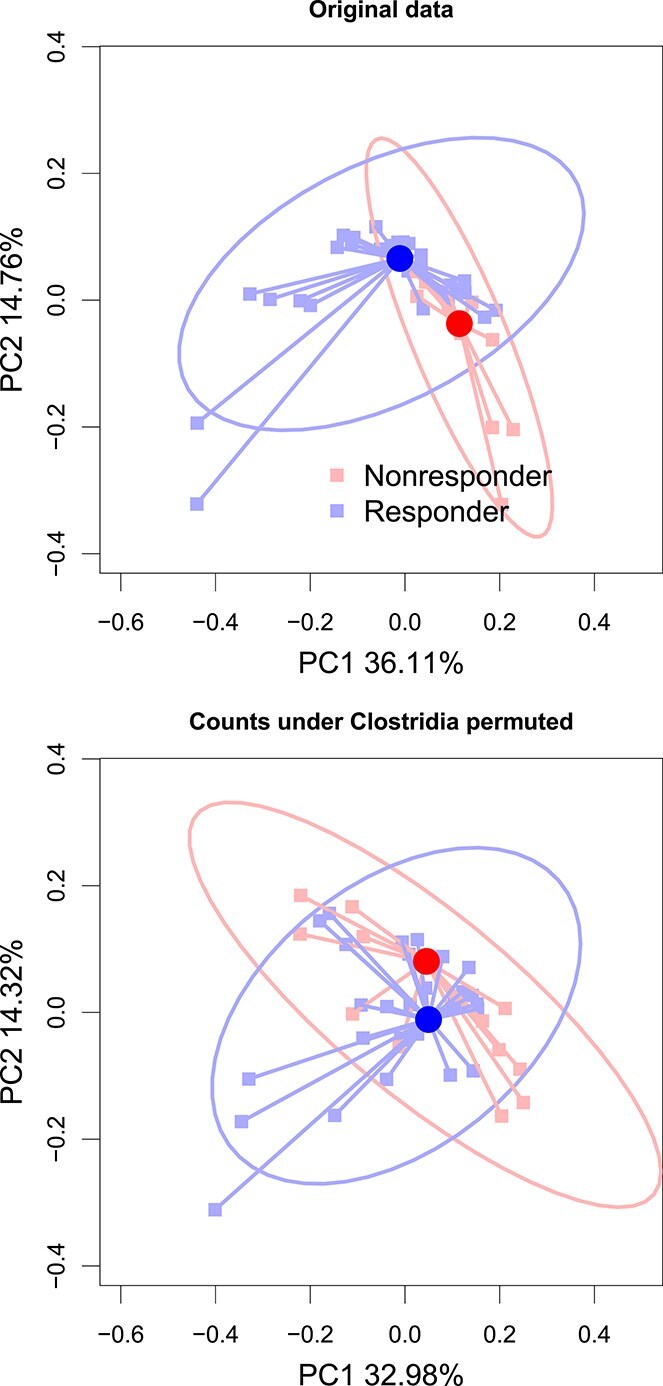
PCoA plots illustrating the global variation in the microbiome for melanoma patients who responded versus did not respond to immunotherapy [16], where we depict both the original data (top) and the modified data after permuting counts belonging to the class Clostridia, with ellipses representing the 95% confidence regions.

We begin with an illustration highlighting the motivation behind our approach. In the top panel of Fig. 1, we show a PCoA plot based on weighted UniFrac distance that depicts variation in microbiome composition between melanoma patients that responded to immunotherapy versus those that did not. The 95% confidence regions for each group indicate there may be global differences in the microbiome profiles between the groups. In the bottom panel, we artificially permuted counts belonging to the class Clostridia; the two clusters of patients become less separated in the PCoA plot. Permuting counts belonging to Clostridia from the data weakened the global association between the microbiome and the response, suggesting that Clostridia may play an important role in driving the global association results. In the remainder of this section, we describe our proposal for a formal testing procedure aimed at quantitatively describing this phenomenon.

In our proposed approach, we start with the finest resolution features, corresponding to the leaf nodes in the taxonomic tree. For microbiome data derived from profiling of the 16S rRNA gene, these features are typically defined as amplicon sequence variants (ASVs) or operational taxonomic units (OTUs). Our method may be applied as well to features derived from whole metagenome sequencing (WGS). By comparing the representative sequence for each feature against an established reference library, the feature can be assigned a taxonomic classification. Taxonomic levels from broad to specific follow the sequence *kingdom*, *phylum*, *class*, *order*, *family*, *genus*, and *species*. Based on the taxonomic assignments, one can draw a taxonomic tree reflecting the relatedness of all the features in the dataset.

Most existing methods for identifying individual feature associations from WGS or 16S data focus on marginal associations and do not quantify how much individual features contribute to the results from global association testing. To fill this gap, we propose **CAT**, which tests the association between specific features and outcomes while conditioning on the tree structure and the abundance of other features in the tree. The **CAT** method is rooted in the coefficient of determination $R^{2}$ for global microbiome association tests. **CAT** estimates the change in $R^{2}$ for a global test using the original dataset versus modified datasets with the counts under the taxon of interest permuted, and obtains the empirical $P$-value. One widely used global association test is the nonparametric PERMANOVA method [7, 18]. We begin by briefly reviewing this test, which serves as a starting point for our proposed method.

Consider the $n \times n$ matrix of pairwise distances between $n$ observations $\mathbf{D}=[d_{ij}],$ where $d_{ij}$ represents the distance between observation $i$ and observation $j.$ We transform $\mathbf{D}$ to a new matrix $\mathbf{A}=[a_{ij}]=[-\frac{1}{2}d_{ij}^{2}]$, and center $\mathbf{A}$ to get Gower’s centered matrix 


\begin{align*}& \mathbf{G} = \left(\mathbf{I}-\frac{\mathbf{11^{\prime}}}{n}\right)\mathbf{A}\left(\mathbf{I}-\frac{\mathbf{11^{\prime}}}{n}\right),\end{align*}

where $\mathbf{I}$ represents the $n \times n$ identity matrix, and $\mathbf{11^{\prime}}$ represents an $n \times n$ matrix of all 1s. With an $n \times g$ design matrix $\mathbf{X}$ providing information on $g$ covariates, we can compute the hat matrix $\mathbf{H}=\mathbf{X}^{\prime}(\mathbf{X}^{\prime}\mathbf{X})^{-1}\mathbf{X}$. From the hat matrix, we can further calculate the total sum-of-squares ($SS_{T}$), the among-group sum-of-squares ($SS_{A}$), and the residual sum-of-squares ($SS_{R}$) as in MANOVA: 


\begin{align*} &SS_T=\mathrm{tr}(\mathbf{G}), \quad SS_A=\mathrm{tr}(\mathbf{HG}), \textrm{ and} SS_R=\mathrm{tr}\big[(\mathbf{I}-\mathbf{H})\mathbf{G}\big].\end{align*}

Just as in MANOVA, the coefficient of determination $R^{2}$ can be calculated as the ratio of the sum of squares between groups ($SS_{A}$) to the sum of squares total ($SS_{T}$). It provides an indication of the strength of the relationship between the outcome variable and the microbiome profiles, with a value closer to 1 indicating a stronger relationship.

We now describe how to apply **CAT** to test the conditional association between the outcome of interest and a specific taxon. Let $X$ denote the outcome vector for $n$ observations. Let $\mathbf{Z}$ represent the $n \times m$ matrix with the observed counts for the finest-resolution microbiome features, which correspond to the leaf nodes in a taxonomy tree $\mathcal{T}$ with $m$ leaves. We denote the set of leaf nodes for the full tree $\mathcal{L}(\mathcal{T}) = \{1, \ldots , m\}$. For any internal node in the tree $t$, we let $\mathcal{L}(t)$ denote the leaf nodes corresponding to its descendants. Given these definitions, we lay out the steps of the **CAT** procedure as follows:

1. Calculate the $n \times n$ sample pairwise distance matrix $\mathbf{D}$ for the original data matrix $\mathbf{Z}$.
2. Perform PERMANOVA using $\mathbf{D}$ and $X,$ and obtain a coefficient of determination $R^{2}$ for the outcome of interest.
3. For the specific taxon $t$ being tested by **CAT**, generate $B$ new data matrices $\mathbf{Z}^{b},$  $b=1,\dots , B$ by permuting elements of $\mathcal{L}(t)$ by column.
4. Calculate $B$ new pairwise distance matrices $\mathbf{D}^{b}$ using the modified data matrix $\mathbf{Z}^{b}$.
5. Perform PERMANOVA using $\mathbf{D}^{b}$ and get new coefficients of determination $R^{2}_{b}$ for the outcome of interest.
6. Estimate the $P$-value as the proportion with an increasing coefficient of determination: $\hat{p}=\frac{1}{B}\sum _{b=1}^{B} I(R^{2}-R^{2}_{b}<0).$

**Figure 2:**
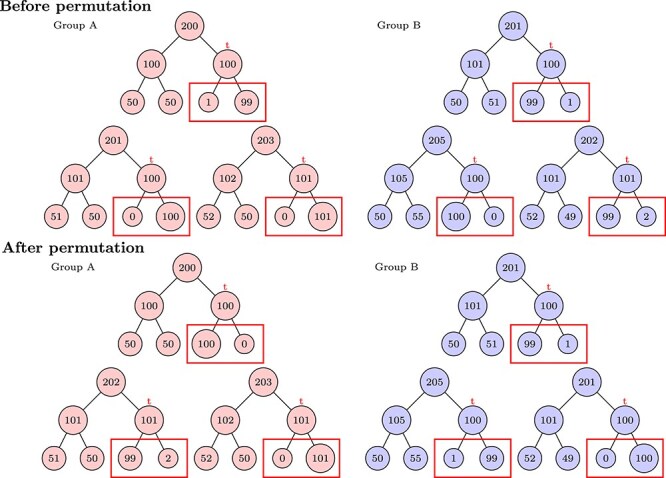
A schematic plot showing how to permute the descending counts for a particular taxon, where taxa in the red rectangles are children of the taxon of interest.

Figure S7 provides a flowchart of the **CAT** procedure. Figure 2 provides a toy example to illustrate how **CAT** permutes the leaf counts under a specific taxon $t$ (marked in red) in Step 3 of the procedure. Suppose that taxon $t$ is strongly associated with the outcome of interest and that this association is not captured by other features in the tree. In that case, permuting the counts descending from $t$ (marked by red boxes) will decrease the $R^{2}$ in the PERMANOVA test. In contrast, permuting a non-discriminating taxon would minimally affect the $R^{2}$ of the PERMANOVA test.

### Conditional testing for MiRKAT

The MiRKAT method [9], which has been extended to handle survival outcomes [10] and correlated or dependent samples [11, 12], offers a powerful global association test based on the kernel regression framework.

For the simplest situation where the outcome of interest $X$ is continuous, the model can be expressed as 


\begin{align*} & x_i=\beta_0+\boldsymbol{\beta}\mathbf{u}_i+f(\mathbf{z}_i)+\varepsilon_i, \quad i=1,2,\dots,n, \end{align*}

where $x_{i}$ is the outcome for the $i$th subject, $\beta _{0}$ is the intercept term, $\boldsymbol{\beta }$ is a vector of regression coefficients, and $\mathbf{u}_{i}$ is a vector of covariates unrelated to the microbiome. The microbiome information for the $i$th sample is characterized by $\mathbf{z}_{i}$, and $f(\mathbf{z}_{i})$ is the output from a reproducing kernel Hilbert space $\mathcal{H}_{k}$. The microbiome association test is equivalent to testing $f(\mathbf{z})=0$. The MiRKAT test statistic is derived from a variance component score test within a linear mixed model, and it is defined as 


\begin{align*} &Q = \frac{(X - \hat{\mu}_0){\prime} \mathbf{G} (X - \hat{\mu}_0)}{2\phi},\end{align*}

where $\mathbf{G}$ is the Gower’s centered matrix as in PERMANOVA and $\phi $ is equal to the estimated residual variance under the null hypothesis. $\hat{\mu }_{0} = (\hat{\mu }_{0,1}, \ldots , \hat{\mu }_{0,n})$ are the fitted values under the null hypothesis of no microbial effect on the outcome, computed as $\hat{\mu }_{0,i}= \hat{\beta }_{0}+\widehat{\boldsymbol{\beta }}\mathbf{u}_{i}.$

Zhan [19] showed that the squared MiRKAT statistic is proportional to the coefficient of determination $R^{2},$ up to a constant factor. In this setting, $R^{2}$ characterizes the fraction of variability in outcome similarity explained by microbiome similarity. This permits the use of **CAT** for testing conditional association for a particular taxon. To implement this approach, the MiRKAT procedure can replace PERMANOVA in Steps 2 and 5 of the **CAT** procedure. When multiple distance metrics are used, users can take the maximum of the $R^{2}$ from different metrics. More broadly, any valid global testing method can be used in these steps.

In PERMANOVA, when multiple covariates are present, we can compute the ratio between the sum of squares attributed to the covariate of interest and the total sum of squares. When paired with MiRKAT, the effects of other covariates are already accounted for through the term $\boldsymbol{\beta }\mathbf{u}_{i},$ thus there is no further need for adjustments.

## Simulation study

In this section, we illustrate the utility of **CAT** as a follow-up to global testing and compare results from **CAT** with those from existing marginal testing approaches on simulated data. We develop realistic simulation scenarios by starting from a real microbiome data set, which will be examined more closely in Section “Application to real data.” We adopt a “spike-in” method to control the taxa driving the cross-group differences.

### Data generation

To construct our simulation, we first obtained the 16S sequencing data from Gopalakrishnan *et al*. [16], which examined the association between the gut microbiome and response to immunotherapy in melanoma patients. This is the same data set illustrated in Fig. 1. The original data included $43$ patients; $30$ responded to therapy, while the rest were non-responders. The sequencing depth per sample in this study had a mean of 48,765. To quantify features from the raw sequencing data, we applied the UNOISE2 function [20] to the 16S rRNA gene sequences, identifying 1455 ASVs. Given the sequence for each ASV, we then applied the FastTree algorithm [21] to build a phylogenetic tree. FastTree employs an approximate maximum-likelihood framework, utilizing heuristics to constrain the search for tree topologies. By doing so, it circumvents exhaustive search processes while maintaining a high level of accuracy. This efficiency makes FastTree well-suited for large-scale microbiome datasets.

In our simulation set-up, we assume there are two groups (group 0 and group 1), each with 31 observations. We use the mean sequencing depth of the melanoma data as the number of sequences for each sample. The steps for generating the simulated data are as follows:

1. For each group, set the expected abundance of each microbiome feature to that of the marginal distribution of the Gopalakrishnan *et al*. [16] dataset.
2. Generate the number of sequences for each ASV from a Dirichlet multinomial distribution with the sum of parameters for the Dirichlet distribution set to $62$. (In the Gopalakrishnan *et al*. [16] dataset, if we assume the data are from two Dirichlet multinomial distributions, the sums of the parameters are estimated to be $70$ for the responder group and $54$ for the nonresponder group.)
3. For group 1, add a random number generated from a Poisson distribution with parameter $\lambda $ to the number of sequences for the ASVs belonging to the feature being “spiked-in.”

Varying the parameter $\lambda $ affects the signal strength; we test the performance of our method with $\lambda $ set to 5, 10, 30, 50, and 70. The simulation study has two scenarios corresponding to two differential features: family Porphyromonadaceae, which accounts for 32 ASVs and 3.5% of the sequences in the dataset; and family Lachnospiraceae (346 ASVs, 14.0% of sequences). We chose moderately abundant families to best illustrate different performances across methods. The range of family abundances varies widely and is highly skewed, with Bacteroidaceae comprising the highest proportion ($41.98\%$) and Aeromonadaceae the lowest ($0.00005\%$), resulting in a median abundance of $0.01\%.$ In order to assess type I error control, we include the setting without any “spike-in,” which corresponds to the null hypothesis of no difference between groups.

### Methods compared

In applying **CAT**, we set the number of permutation samples to 200. To fully leverage the phylogenetic information, we use the weighted UniFrac distance, which accounts for ASV abundance, as well as the topology and branch lengths of the phylogenetic tree. The phylogenetic tree used for calculating the weighted UniFrac distances is the phylogenetic tree of Gopalakrishnan *et al*. [16]’s dataset. To illustrate the utility of **CAT** as a follow-up to global hypothesis testing, we show the distribution of $R^{2}$ values in the original and modified data sets as side-by-side boxplots. Although **CAT** is unique in its focus on conditional association testing, we also provide results from the following marginal testing methods: a basic Mann–Whitney test, bias-corrected ANCOM(ANCOM-BC) [15], and DESeq2 [14]. Both the original and adjusted $P$-values for the DESeq2 method were computed. However, these methods have a different null hypothesis than **CAT**, so cannot be considered as direct competitors.

**Figure 3:**
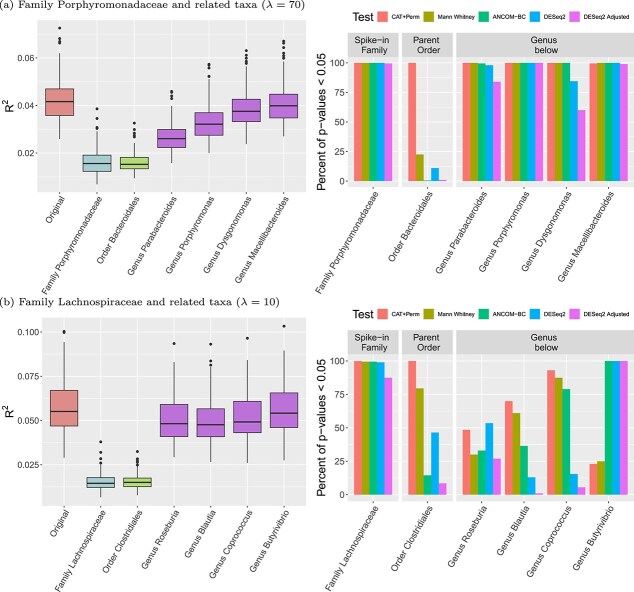
Boxplots of the $R^{2}$ values (left) across 200 simulations and barplots of the percentage of $P$-values <.05 from **CAT**, Mann–Whitney, ANCOM-BC, and DeSeq2 (right) over 200 simulated datasets, where the first panel in each subplot in the right column represents results from the manipulated family, followed by the order above and the child genera below.

### Results

Here, we report findings from the **CAT** test paired with PERMANOVA using the weighted UniFrac metric with (simulation parameter reflecting signal strength) $\lambda =70$ for Porphyromonadaceae and $\lambda =10$ for Lachnospiraceae across 200 simulated datasets. We chose to focus on these settings as all methods achieved high power to detect the spiked-in feature, but had differential results regarding the significance of its parent and child nodes. The results from **CAT** for other $\lambda $ values can be found in the Supplemental Material. Permuting sequences from the spiked-in feature or its parent node results in a sharp reduction in coefficient of determination $R^{2}$ values for both synthetic data sets (Fig. 3 at left). The effect of permuting sequences from the child nodes is more nuanced; in the first data set, child nodes of Porphyromonadaceae are responsible for explaining some portion of the $R^{2}$ value, while the child nodes of Lachnospiraceae may contribute less independent information. The percent of $P$-values $< 0.05$ for the **CAT** test along with the results from existing marginal tests are shown at right in Fig. 3. In addition to the family being “spiked-in,” i.e. the feature with an abundance difference constructed between the groups under the simulation design, we also offer the hypothesis testing results for the order above and the genera below. The proposed **CAT** method can correctly reject the null hypothesis for the family directly manipulated and the order above. In some cases, the $P$-values obtained from **CAT** are congruent with those obtained from marginal tests. However, for some hypotheses the conditional testing approach of **CAT** is relatively more conservative than marginal tests. In particular, the genus *Butyrivibrio*, a child of the spiked-in family Lachnospiraceae, is consistently found to be significant by ANCOM-BC and DESeq2, while the **CAT** results do not consistently indicate this feature as a driver of the global association results. This behavior of **CAT** reflects the nature of the conditional test; it will be less likely to reject the null hypothesis when other features have already explained the cross-group differences. In contrast, other tests estimate the marginal effect, which may overemphasize the importance of lower-level taxa.

**Figure 4:**
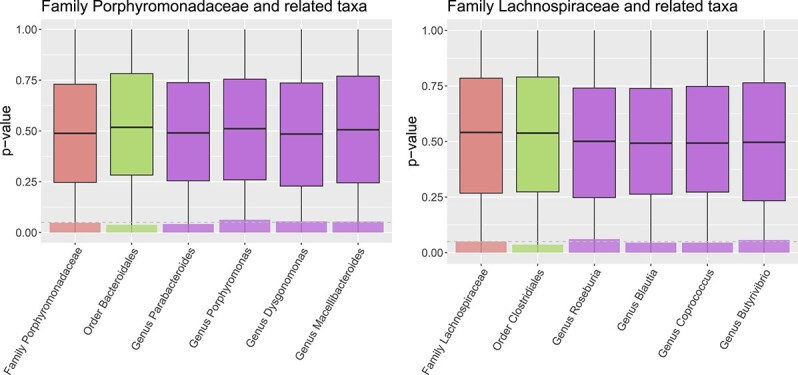
Simulation results for null scenario without “spike-in”: $P$-values (boxplots) and proportion of $P$-values $<.05$ (transparent bars), with the targeted significance level of 0.05 shown as a dashed line.

To illustrate the behavior of **CAT** under the null, we also provide results without “spike-in.” Figure 4 illustrates that in the absence of a signal, the distribution of the $P$-values is centered around 0.5, with a wide spread across the unit interval. In Fig. 4, we also represent the proportion of $P$-values $< 0.05$ using semitransparent bars. Notably, the proportion of false rejection is close to 0.05, suggesting type I error inflation is not a concern for the proposed method.

## Application to real data

We now use **CAT** to analyze two real datasets examining the role of the microbiome in shaping melanoma patient outcomes. First, we consider the data set from Gopalakrishnan *et al*. [16], which dealt with the association of the microbiome to immunotherapy response. Later, we apply **CAT** to the study of Spencer *et al*. [22], which characterized the role of the microbiome in shaping progression-free survival (PFS).

### Binary response

In this data set, there are global differences in microbiome composition between patients that responded to immunotherapy versus those that did not; the $P$-value from the PERMANOVA test using weighted UniFrac for responder versus nonresponder is <0.001. To identify associated features, Gopalakrishnan *et al*. [16] relied on LEfSe [23], the first step of which is to screen features with a Mann–Whitney test. We applied **CAT** to ascertain whether the hits they identified using LEfSe remained significant under a conditional test. In addition to the binary response, we also have information on whether the patient received previous targeted therapy. Moreover, we applied the **CAT** method using Bray–Curtis distance. The results are presented in Supplemental Table S1.

**Figure 5:**
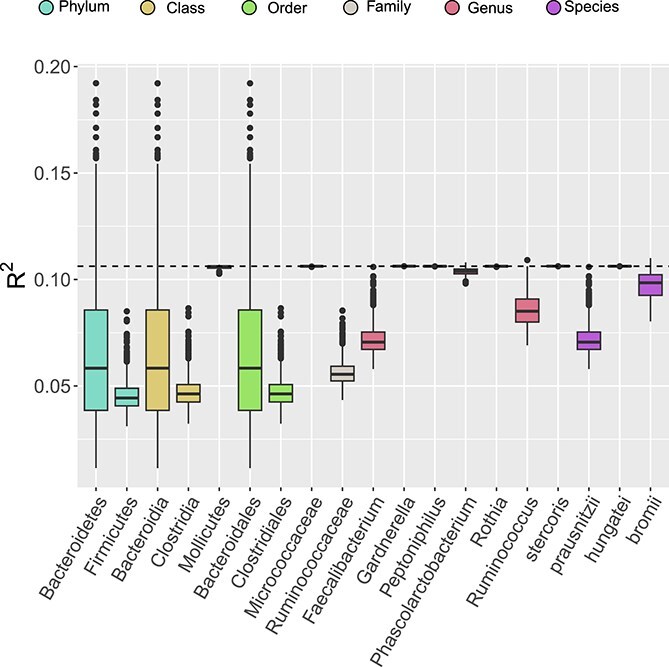
Boxplots of $R^{2}$ values from the application of **CAT** to Gopalakrishnan *et al*. [16]’s dataset described in Subsection “Binary response,” where the dashed line indicates the original $R^{2}$ of the dataset.

Table 1 shows the Mann–Whitney $P$-values and $P$-values obtained using **CAT**; significant hits from **CAT** include the Phylum Firmicutes, its subordinate class Clostridia, the order Clostridiales under Clostridia, the family Ruminococcaceae within Clostridiales, and the genus *Ruminococcus* that comes under Ruminococcaceae. Additionally, the species *R. bromii* beneath *Ruminococcus* is deemed significant. Within the same family Ruminococcaceae, the genus Faecalibacterium and the species underneath, prausnitzii, are also significant. However, some hits that were found to be significant using LEfSe, including the genus *Gardnerella* and the species *B. stercoris* lose significance; this suggests that these microbiome features might not be good candidates for a microbiome intervention. The $R^{2}$ values obtained by permuting counts across different taxa are shown in Fig. 5. Specifically, some taxa—such as the abundant phylum Bacteroidetes, the class Bacteroidia, and the order underneath it, Bacteroidales—tend to have smaller $R^{2}$ values, although the ranges of $R^{2}$ for permuted datasets are wide. This suggests that their abundance has a heterogeneous effect on the population. In contrast, other taxa—including the class Mollicutes, the family Micrococcaceae, the genus *Gardnerella*, the genus *Rothia,* and the species *B. stercoris*—have permuted $R^{2}$ values that are close to the original dataset’s, indicating that after accounting for phylogenetic information, these taxa appear to have only a minor influence on the outcome of interest. Overall, **CAT** provides many results that are congruent with the original paper, which uses LefSe with a Mann–Whitney test as its first step, yet provides novel insights into the conditional association.

**Table 1:** Levels in the taxonomic tree, taxa, Mann–Whitney(MW) $P$-values, and **CAT**$P$-values when applied to features identified by LEfSe in Gopalakrishnan *et al*. [16]

Level	Taxon	MW	**CAT**
		$P$ -value	$P$ -value
Phylum	Bacteroidetes	$\mathbf{<0.01}$	$0.126$
Phylum	Firmicutes	$\mathbf{<0.01}$	$\mathbf{<0.001}$
Class	Bacteroidia	$\mathbf{<0.01}$	$0.126$
Class	Clostridia	$\mathbf{<0.01}$	$\mathbf{<0.001}$
Class	Mollicutes	$\mathbf{0.01}$	$0.358$
Order	Bacteroidales	$\mathbf{<0.01}$	$0.126$
Order	Clostridiales	$\mathbf{<0.01}$	$\mathbf{<0.001}$
Family	Micrococcaceae	$\mathbf{0.01}$	$0.262$
Family	Ruminococcaceae	$\mathbf{0.03}$	$\mathbf{<0.001}$
Genus	*Faecalibacterium*	$\mathbf{0.01}$	$\mathbf{<0.001}$
Genus	*Gardnerella*	$\mathbf{0.03}$	$0.983$
Genus	*Peptoniphilus*	0.12	$0.134$
Genus	*Phascolarctobacterium*	$\mathbf{0.01}$	$0.052$
Genus	*Rothia*	$\mathbf{0.01}$	$0.262$
Genus	*Ruminococcus*	$\mathbf{0.03}$	$\mathbf{0.002}$
Species	*B. stercoris*	$\mathbf{0.03}$	$0.966$
Species	*F. prausnitzii*	$\mathbf{0.01}$	$\mathbf{<0.001}$
Species	*M. hungatei*	0.18	$0.268$
Species	*R. bromii*	0.08	$\mathbf{0.042}$

### Survival outcomes

To demonstrate the application of **CAT** for survival outcomes, we employed it to analyze the dataset from Spencer *et al*. [22]. This dataset included 163 subjects undergoing systemic therapy for melanoma that were profiled using 16S rRNA sequencing and followed for PFS. Among these subjects, 86 progression events were observed, with a median PFS of 1.8 years. The microbiome profiling data included 3306 ASVs, corresponding to 346 unique taxa at the genus level or higher. In our case study, we focused on taxa found to be associated with treatment response by Spencer *et al*. [22], including the phylum Firmicutes, class Clostridia, order Oscillospirales, family Ruminococcaceae, and genera *Faecalibacterium* and *Ruminococcus*. We also tested the genera *Bifidobacterium* and *Lactobacillus*, as these are popular in commercially available supplements and were tested as probiotic interventions as part of a preclinical experiment in the same study [22]. To apply **CAT**, we used Bray–Curtis and Jaccard distances with the MiRKAT-S method. In addition, we ran the univariate Cox model for each candidate feature for comparison.

Table 2 displays the outcomes of our **CAT** method. Using a conditional approach, **CAT** identifies the family Ruminococcaceae and the genus *Faecalibacterium* as significant while accounting for the phylogenetic structure, the tree branch lengths, and other coexisting taxa. Similar findings were presented in Li *et al*. [24], where they observed melanoma patients who responded to treatment had elevated levels of the family Ruminococcaceae and the genus *Faecalibacterium* in their gut microbiota. The findings with the conditional test open the door to potential probiotic interventions, as the effects of these taxa persist even after adjusting for the presence of other taxa. In contrast, the marginal association of the genus *Bifidobacterium* does not hold up when we consider the conditional test, suggesting *Bifidobacterium* might not be a good target for intervention.

**Table 2:** Level in the taxonomic tree, taxon, univariate Cox model $P$-value, and $P$-value from **CAT** when applied to features of interest from Spencer *et al*. [22]

Level	Taxon	Cox	**CAT**
		$P$ -value	$P$ -value
Phylum	Firmicutes	$0.54$	$0.2095$
Class	Clostridia	$0.46$	$0.1855$
Order	Oscillospirales	$0.18$	$0.0825$
Family	Ruminococcaceae	$\mathbf{0.04}$	$\mathbf{0.0195}$
Genus	*Faecalibacterium*	$\mathbf{0.02}$	$\mathbf{0.0115}$
Genus	*Ruminococcus*	$0.31$	$0.7270$
Genus	*Bifidobacterium*	$\mathbf{0.02}$	$0.2335$
Genus	*Lactobacillus*	$0.58$	$ 0.2490$

In addition to 16S sequencing data with a moderate number of observations, the method can also be applied to large-scale whole-genome shotgun (WGS) sequencing data. As an example, we demonstrate the application of the method to the Zeevi *et al*. [25] dataset, which includes 900 samples, in the Supplemental material.

## Concluding remarks

To date, most existing methods for microbiome association testing have focused on either global or marginal testing. Here, we adopt a conditional testing framework, proposing the **CAT** method as a conditional association test using a permutation approach. **CAT** combines the classic permutation idea with the flexibility of using various metrics and testing approaches designed for microbiome data. It is worth mentioning that though microbiome data motivate us to propose **CAT**, the method is widely applicable to a wide range of situations where non-Euclidean pairwise distances are used. In this paper, we only illustrate how to test the conditional association for one taxon; testing the effect of several taxa as a unit is also possible in Steps 3 and 5 of the procedure.

Although our simulation results show that **CAT** may identify fewer features than marginal tests, particularly at lower levels in the tree, we do not directly address multiple testing in this paper. Commonly used multiplicity adjustments, such as the Bonferroni procedure or Benjamini–Hochberg procedure, can be applied to $P$-values generated by **CAT**. However, the null hypotheses under testing in microbiome data sets are not independent. False discovery rate control in correlated conditional tests, particularly in the presence of a phylogenetic tree structure, is an area that we plan to address in the future.

The results from the **CAT** method have clear real-world relevance. There is a growing effort to develop interventions aimed at reshaping the microbiome by administering “rationally designed” mixtures of bacterial strains [26], which have been selected to confer potential benefit to the patient. Our method can identify potentially influential features conditioned on other bacteria’s existence. This will help clinicians identify intervention targets more efficiently.

**Key Points**

- CAT tests a taxon’s conditional association while accounting for other taxa and the phylogenetic structure.
- By focusing on each taxon’s added explanatory value, **CAT** reduces nested discoveries and pinpoints the most informative features for microbiome interventions.
- By pairing with global association tests, **CAT** can handle various types of outcomes, including continuous, binary, correlated, and survival outcomes.

## Supplementary Material

supplemental_bbaf326

## Data Availability

Our method has been implemented in the R package CATMicrobiome, which is publicly available at https://github.com/YushuShi/CATMicrobiome.
