# Supplementary material for: CAT: a conditional association test for microbiome data using a permutation approach
Source: Brief Bioinform. 2025 Jul 11;26(4):bbaf326. doi: 10.1093/bib/bbaf326 (PMC12362256; doi:10.1093/bib/bbaf326)
Supplement: supplemental_bbaf326 [file supplemental_bbaf326.pdf]

Supplement to “CAT: A conditional association test for microbiome data using a permutation approach”

Yushu Shi, Liangliang Zhang, Kim-Anh Do, Robert R. Jenq, and Christine B. Peterson

Additional simulation results

Here, we provide additional simulation results with λ = 5, 10, 30, 50, and 70. For a detailed description of the simulation design and methods compared, please see the “Simulation Study” section of the main manuscript.

$\lambda = 5$

(a) Family Porphyromonadaceae and related taxa

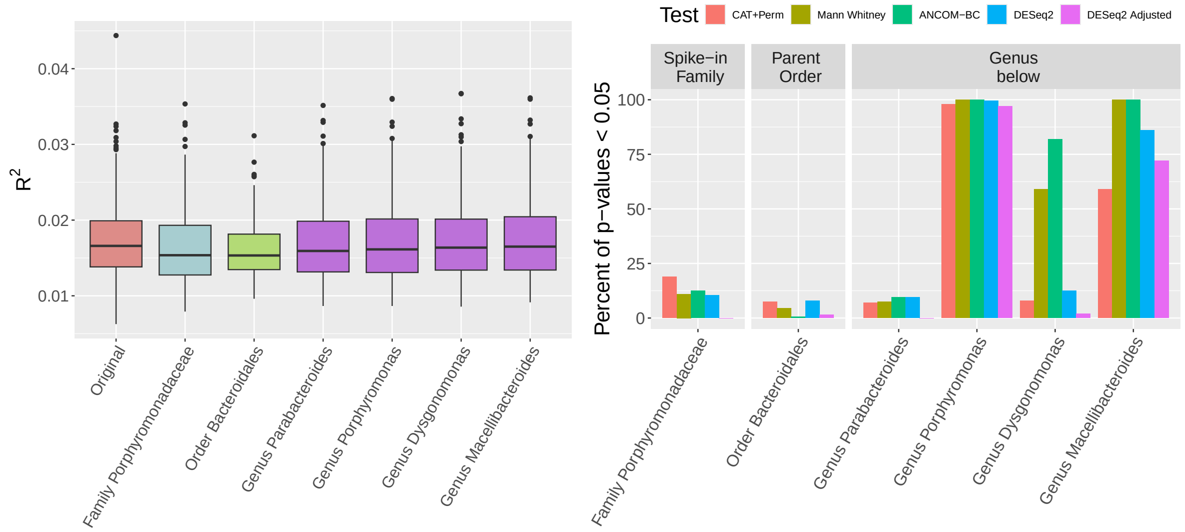

(b) Family Lachnospiraceae and related taxa

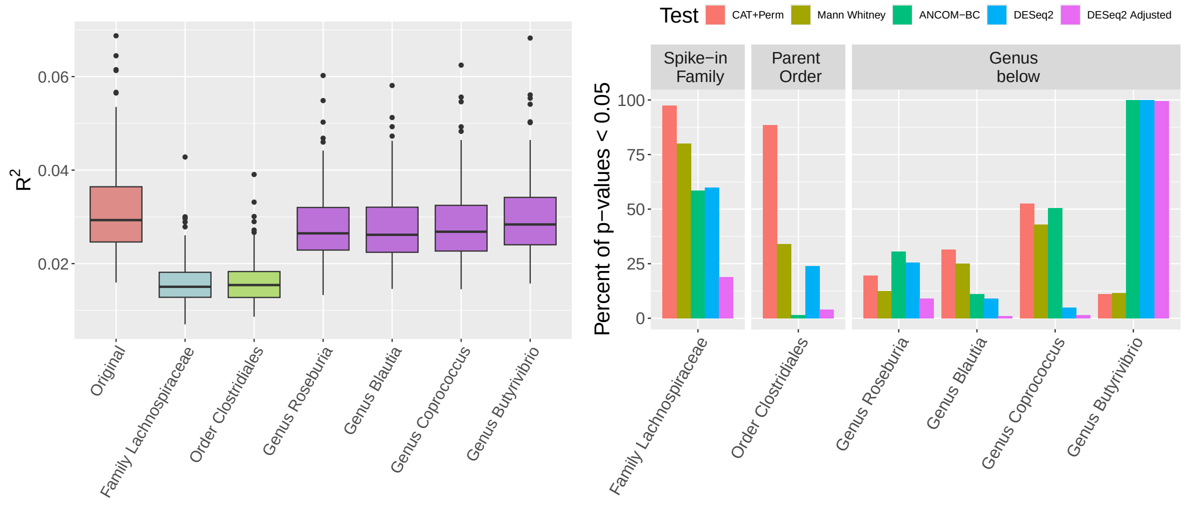

Figure S1: Boxplots of the R^2 values (left) and barplots of the percentage of p-values less than 0.05.

$\lambda = 10$

(a) Family Porphyromonadaceae and related taxa

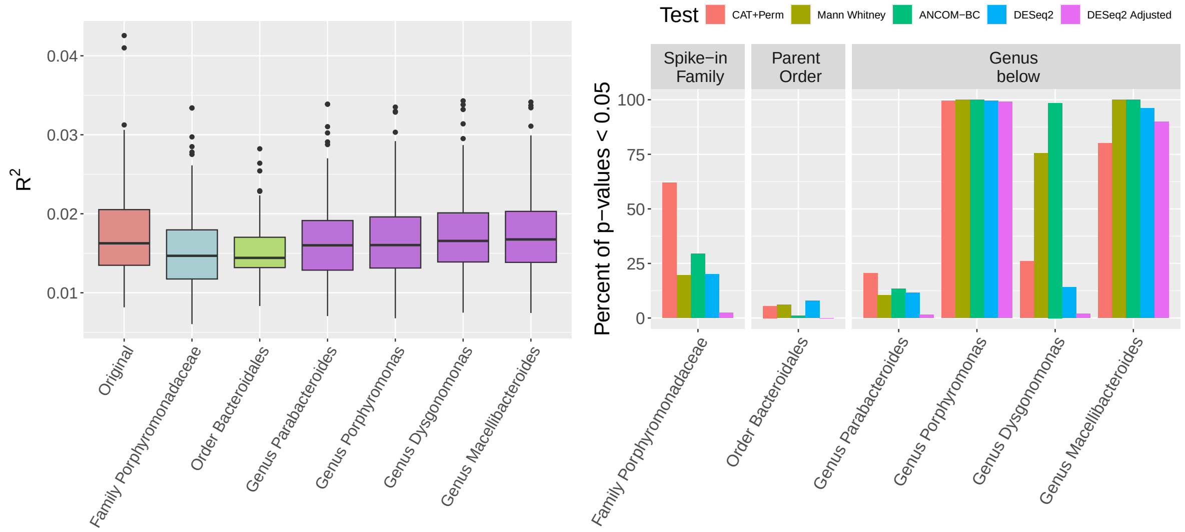

(b) Family Lachnospiraceae and related taxa

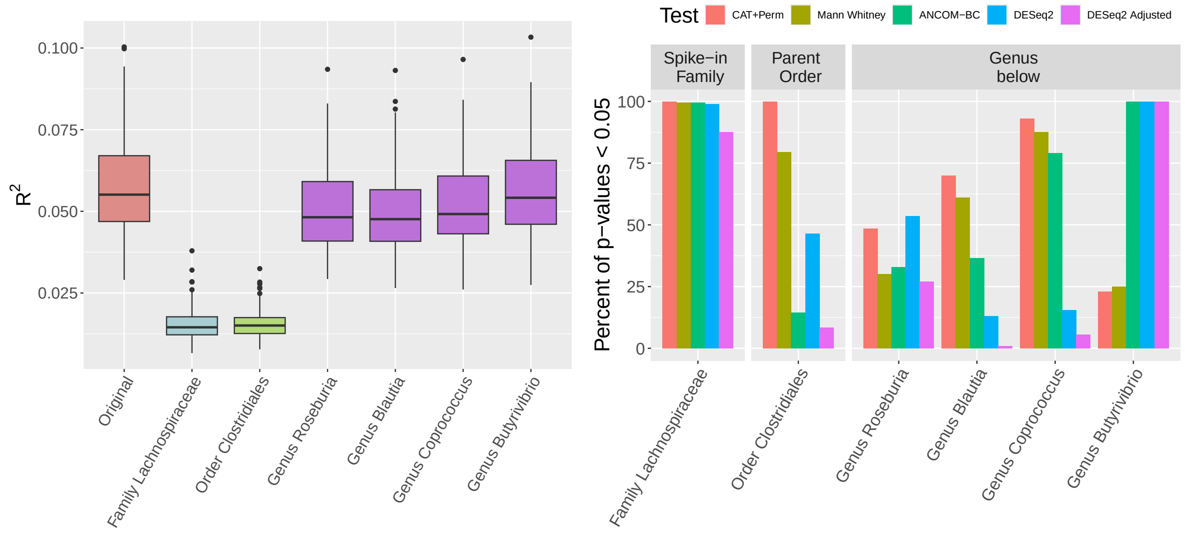

Figure S2: Boxplots of the R^2 values (left) and barplots of the percentage of p-values less than 0.05.

$\lambda = 30$

(a) Family Porphyromonadaceae and related taxa

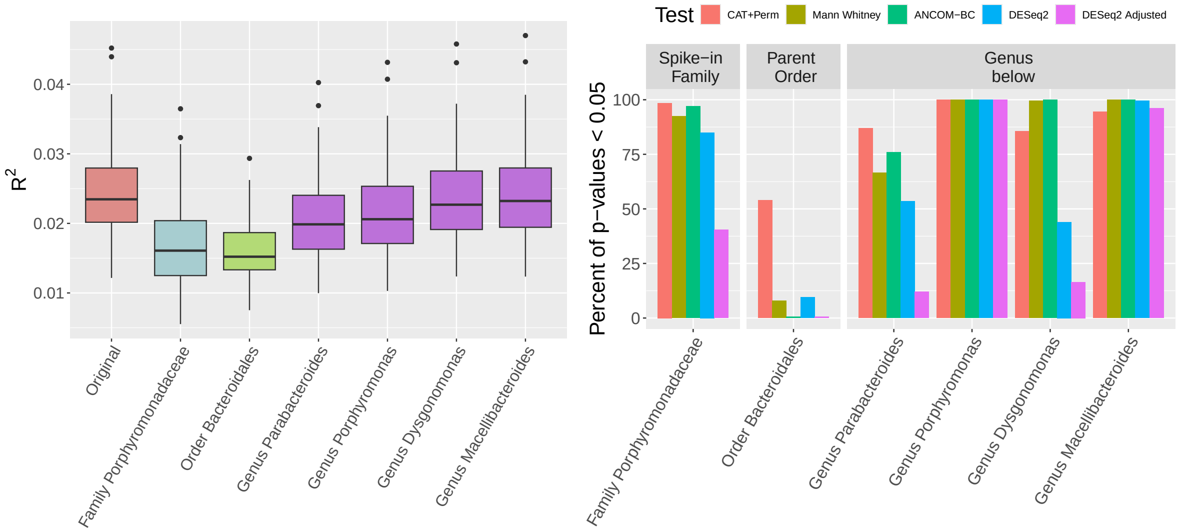

(b) Family Lachnospiraceae and related taxa

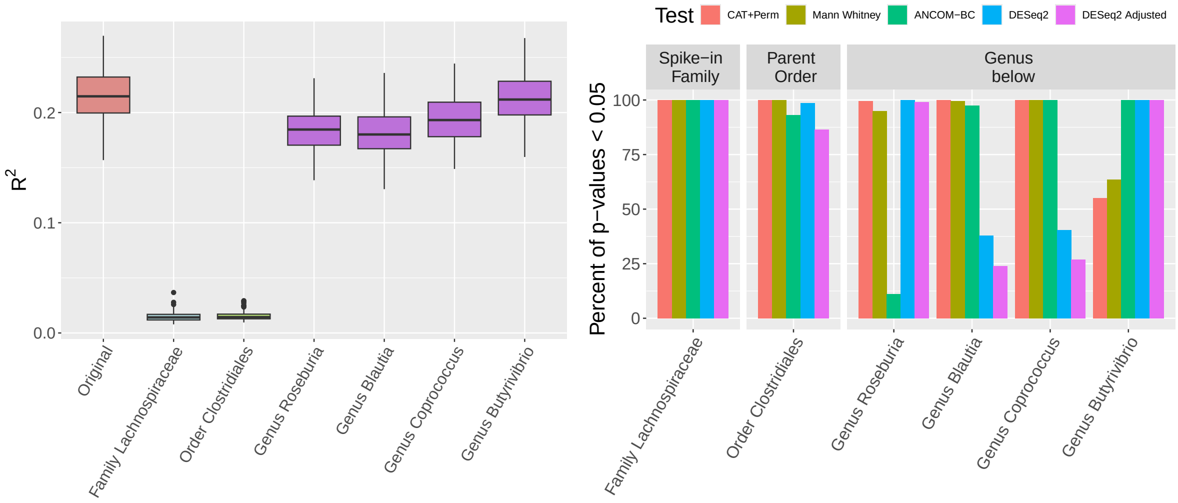

Figure S3: Boxplots of the R^2 values (left) and barplots of the percentage of p-values less than 0.05.

$\lambda = 50$

(a) Family Porphyromonadaceae and related taxa

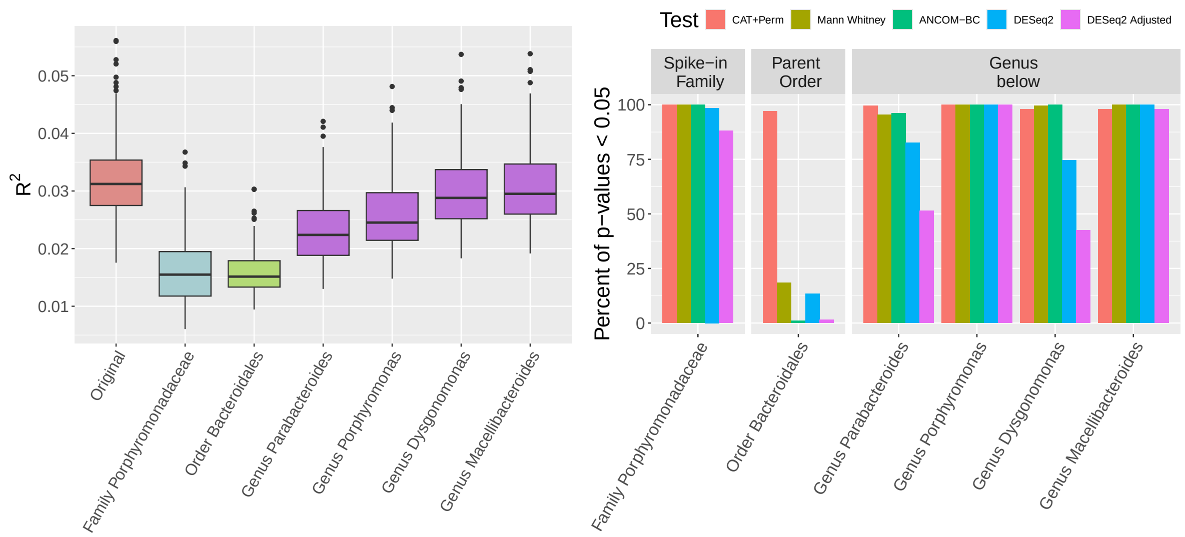

(b) Family Lachnospiraceae and related taxa

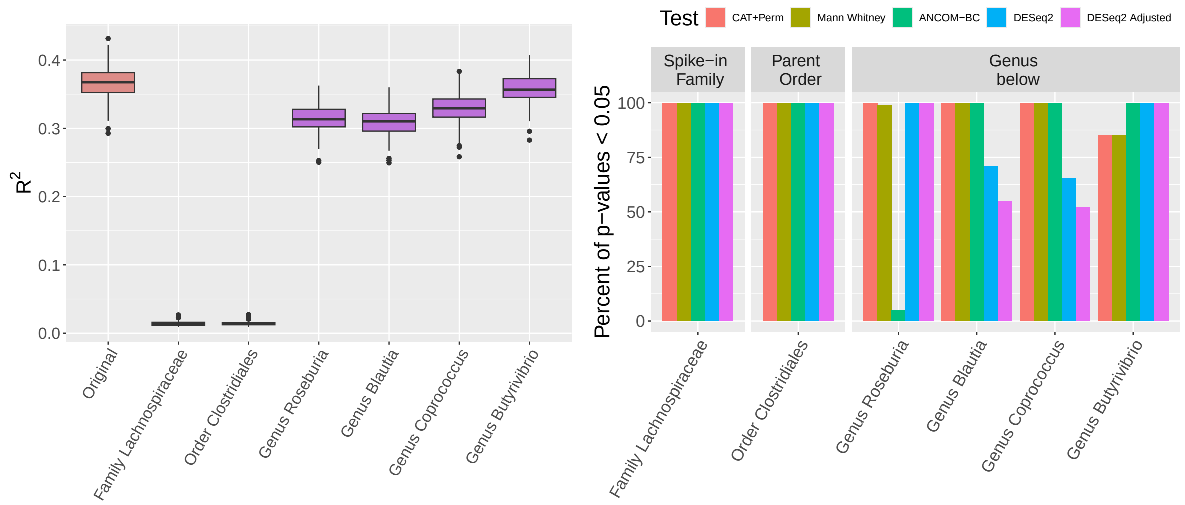

Figure S4: Boxplots of the R^2 values (left) and barplots of the percentage of p-values less than 0.05.

$\lambda = 70$

(a) Family Porphyromonadaceae and related taxa

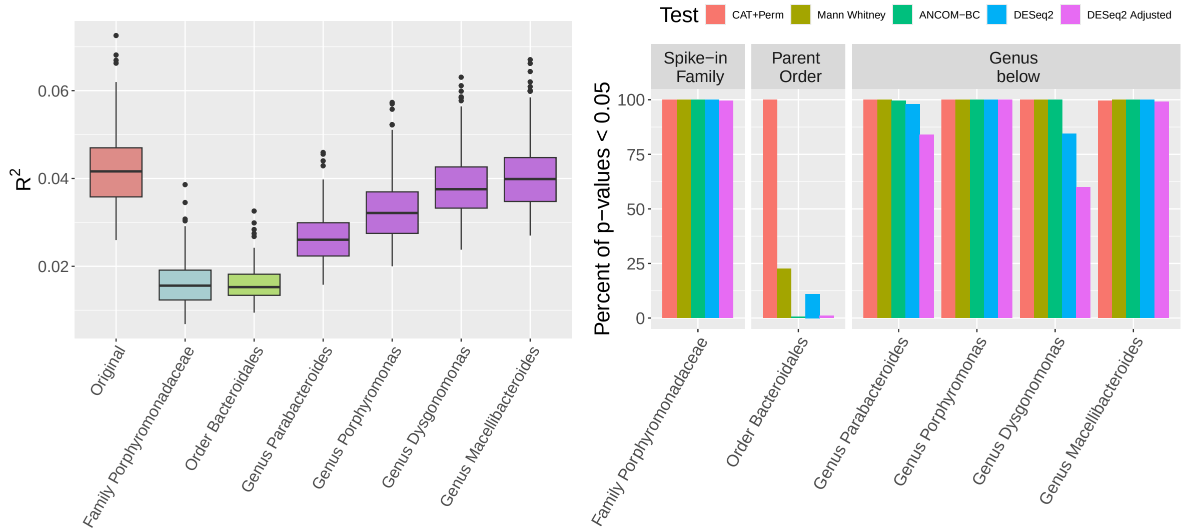

(b) Family Lachnospiraceae and related taxa

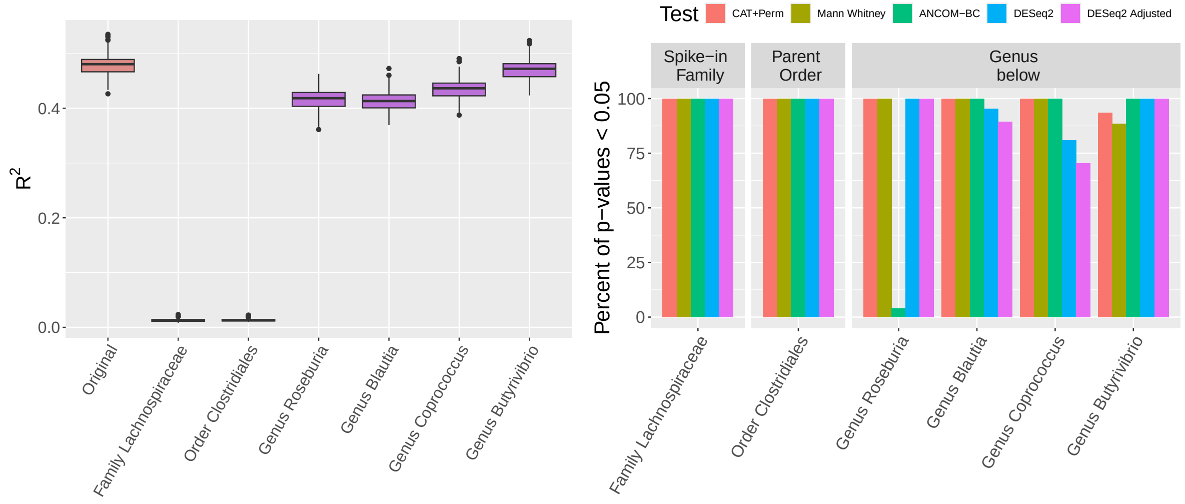

Figure S5: Boxplots of the R^2 values (left) and barplots of the percentage of p-values less than 0.05.

An illustration of the taxonomic and phylogenetic trees

We present the taxonomic and phylogenetic trees for the dataset from Gopalakrishnan et al. [2018]. The left side displays the phylogenetic tree, while the right side shows the taxonomic tree. Notably, the phylogenetic tree has a greater number of branches, as it incorporates evolutionary relationships that are not well captured in the taxonomic tree. Furthermore, the phylogenetic tree reflects branch length information, representing evolutionary distances, which is not encoded in the taxonomic

(a) Phylogenetic tree (b) Taxonomic tree

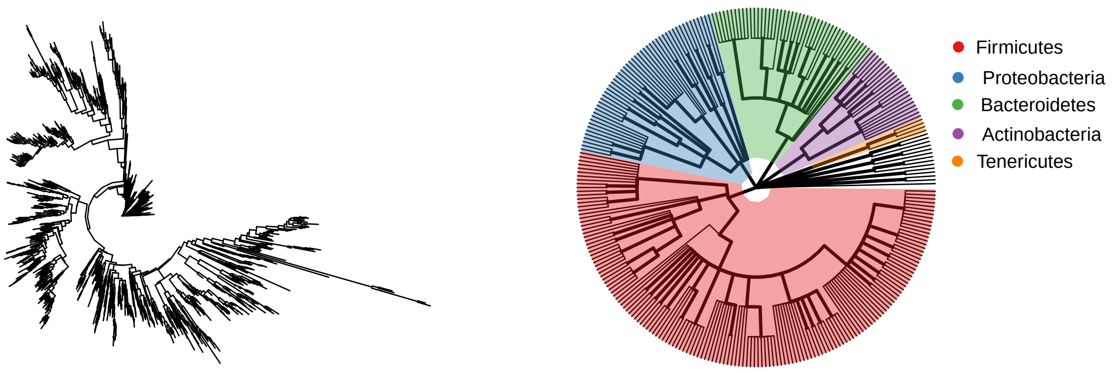

Figure S6: Phylogenetic and taxonomic tree for the Gopalakrishnan et al. [2018] data.

tree. In the taxonomic tree, we use colors to highlight the five most abundant phyla, providing an overview of their relative prevalence—information that is less readily apparent in the phylogenetic tree.

Application to Zeevi et al. [2015] data

The R package curatedMetagenomicData provides gender and BMI information for the participants in the study by Zeevi et al. [2015]. The study includes 900 participants and 673 microbial species. We applied **CAT** using PERMANOVA with Bray-Curtis distances, treating BMI as the outcome while adjusting for gender as a covariate. The number of permutations was set to 1,000. After applying the Benjamini-Hochberg procedure to adjust for multiple testing, we identified three significant taxa at a significance level of $\alpha = 0.05$. These taxa include species CAG-241 from the genus *Oscillibacter*, *A. histaminiformans*, and *S. hippei*.

A flowchart of the CAT method

We provide a flowchart to illustrate the **CAT** method in Figure S7.

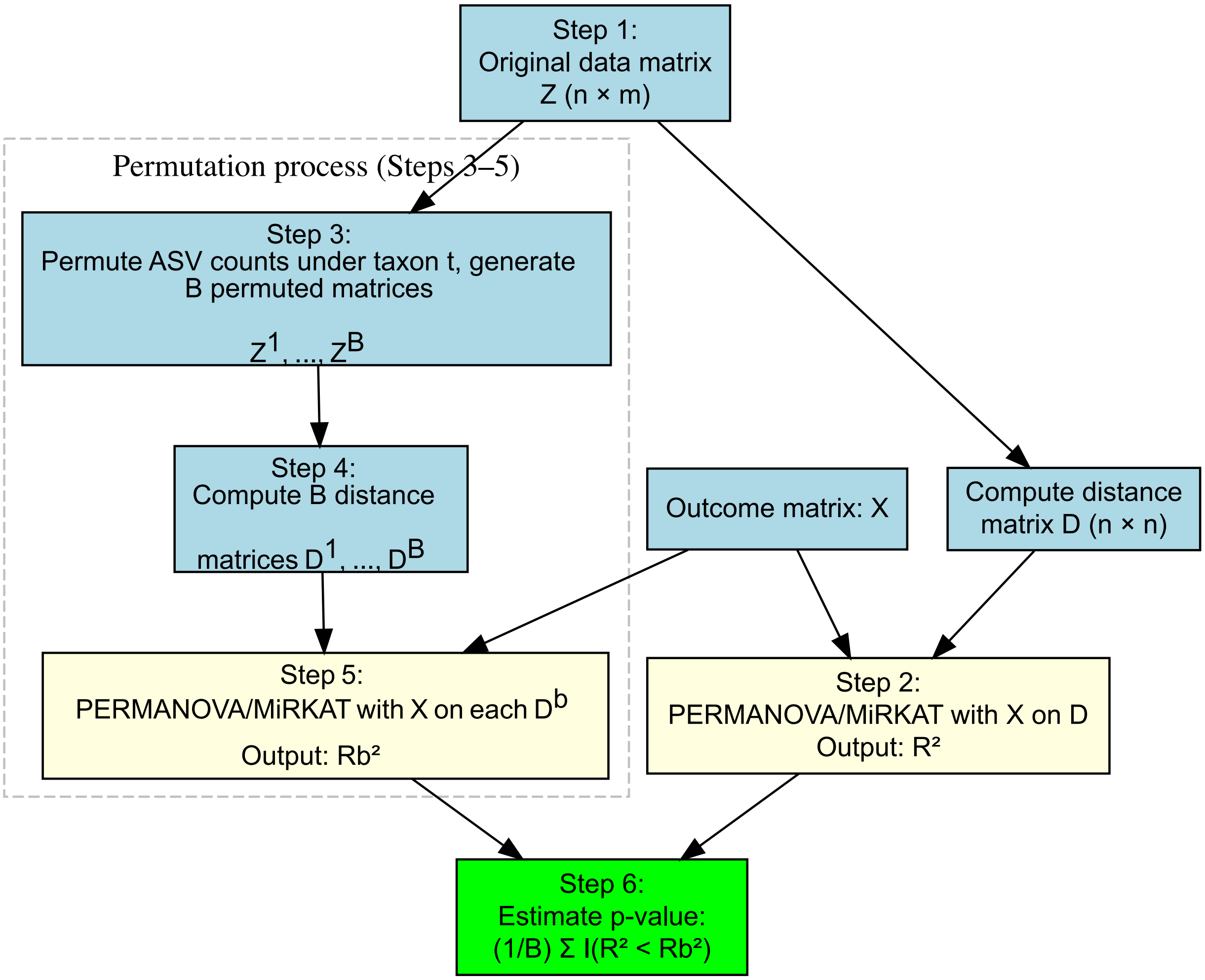

Figure S7: A flowchart of the **CAT** method.

Additional analysis of the dataset from Gopalakrishnan et al. [2018] study

In addition to the binary response, we also have information on whether the patient received previous targeted therapy. We applied **CAT** to identify significant taxa after adjusting for treatment history. Moreover, we applied the **CAT** method using Bray-Curtis distance. The results are presented in Supplemental Table S1. Except for the genus *Phascolarctobacterium* and species *R. bromii*, the significance of other taxa after adjusting for prior targeted therapy remains unchanged, and the p-values for these two taxa are near the 0.05 margin. While the findings for lower taxonomic units are similar, **CAT** with Bray-Curtis distance identified more hits at higher taxonomic levels, including Phylum Bacteroidetes, Class Bacteroidia, and Order Bacteroidales.

Level	Taxon	MW<br>p-value	**CAT**<br>p-value	**CAT** adjusted for<br>prior targeted therapy	**CAT**<br>Bray-Curtis
Phylum	Bacteroidetes	< **0.01**	0.126	0.057	**0.005**
Phylum	Firmicutes	< **0.01**	< **0.001**	< **0.001**	**0.002**
Class	Bacteroidia	< **0.01**	0.126	0.057	**0.005**
Class	Clostridia	< **0.01**	< **0.001**	< **0.001**	**0.002**
Class	Mollicutes	**0.01**	0.358	0.341	0.352
Order	Bacteroidales	< **0.01**	0.126	0.057	**0.005**
Order	Clostridiales	< **0.01**	< **0.001**	< **0.001**	**0.002**
Family	Micrococcaceae	**0.01**	0.262	0.269	0.123
Family	Ruminococcaceae	**0.03**	< **0.001**	< **0.001**	**0.002**
Genus	*Faecalibacterium*	**0.01**	< **0.001**	< **0.001**	**0.031**
Genus	*Gardnerella*	**0.03**	0.983	0.988	0.736
Genus	*Peptoniphilus*	0.12	0.134	0.171	0.256
Genus	*Phascolarctobacterium*	**0.01**	0.052	**0.031**	0.139
Genus	*Rothia*	**0.01**	0.262	0.269	0.123
Genus	*Ruminococcus*	**0.03**	**0.002**	**0.007**	**0.022**
Species	*B. stercoris*	**0.03**	0.966	0.918	0.900
Species	*F. prausnitzii*	**0.01**	< **0.001**	**0.002**	**0.031**
Species	*M. hungatei*	0.18	0.268	0.278	0.176
Species	*R. bromii*	0.08	**0.042**	0.065	**0.044**

Table S1: Levels in the taxonomic tree, taxa, Mann-Whitney (MW) p-values, **CAT** p-values using weighted UniFrac distance, **CAT** p-values adjusted for prior targeted therapy using weighted UniFrac distance, and p-values for **CAT** using Bray-Curtis distance for features identified by LEfSe in Gopalakrishnan et al. [2018].

References

V. Gopalakrishnan, C. Spencer, L. Nezi, et al. Gut microbiome modulates response to anti–PD–1 immunotherapy in melanoma patients. *Science*, 359(6371):97–103, 2018.

D. Zeevi, T. Korem, N. Zmora, et al. Personalized nutrition by prediction of glycemic responses. *Cell*, 163(5):1079–1094, 2025/01/09 2015.
